# Microbubbles Remove *Listeria monocytogenes* from the Surface of Stainless Steel, Cucumber, and Avocado

**DOI:** 10.3390/ma15228203

**Published:** 2022-11-18

**Authors:** Pengyu Chen, Joseph Eifert, Sunghwan Jung, Laura K. Strawn, Haofu Li

**Affiliations:** 1 Department of Food Science and Technology, Virginia Tech, Blacksburg, VA 24061, USA; 2 Department of Biological and Environmental Engineering, Cornell University, Ithaca, NY 14853, USA

**Keywords:** *Listeria monocytogenes*, listeriosis, cavitation bubbles, cross-contamination, cucumbers, avocados

## Abstract

Fresh produce may be contaminated by bacterial pathogens, including *Listeria monocytogenes,* during harvesting, packaging, or transporting. A low-intensity cavitation process with air being injected into water was studied to determine the microbubbles’ efficiency when detaching *L. monocytogenes* from stainless steel and the surface of fresh cucumber and avocado. Stainless steel coupons (1″ × 2″), cucumber, and avocado surfaces were inoculated with *L. monocytogenes* (LCDC strain). After 1, 24 or 48 h, loosely attached cells were washed off, and inoculated areas were targeted by microbubbles (~0.1–0.5 mm dia.) through a bubble diffuser (1.0 L air/min) for 1, 2, 5, or 10 min. For steel, *L. monocytogenes* (48 h drying) detachment peaked at 2.95 mean log reduction after 10 min of microbubbles when compared to a no-bubble treatment. After 48 h pathogen drying, cucumbers treated for 10 min showed a 1.78 mean log reduction of *L. monocytogenes*. For avocados, *L. monocytogenes* (24 h drying) detachment peaked at 1.65 log reduction after 10 min of microbubbles. Microbubble applications may be an effective, economical, and environmentally friendly way to remove *L. monocytogenes,* and possibly other bacterial pathogens, from food contact surfaces and the surfaces of whole, intact fresh produce.

## 1. Introduction

Fresh produce is a significant part of a healthy diet. However, foodborne illness outbreaks have occurred frequently and are increasingly recognized worldwide. This reflects the growing concern regarding safety and hygiene problems in fresh produce. A large number of foodborne outbreaks derive from fresh fruits and vegetables. In 2019, many outbreaks of foodborne illness due to *Salmonella*, *Escherichia coli* O157:H7 *and Listeria monocytogenes* were attributed to the consumption of fruits, seeded vegetables, vegetable row crops, and sprouts in the United States [1].

An important bacterial pathogen that can contaminate fresh produce is *L. monocytogenes*, which can lead to listeriosis. Approximately 1600 people succumb to listeriosis each year, and 260 deaths may occur, based on an estimated report from the U.S. Centers for Disease Control and Prevention [2]. The hospitalization rate of listeriosis can reach 94%. Pregnant women and newborns are high-risk groups for listeriosis [3]. Economically, the annual cost of *L. monocytogenes* control can reach approximately 2.4 billion USD [4].

*L. monocytogenes* can be found on many fresh fruits and vegetables. Additionally, *L. monocytogenes* can be found in a variety of environments, including soil, water, other food products, humans, and animals [5]. Fresh produce such as raw vegetables can be contaminated by *L. monocytogenes* from manure used as soil amendments. Additionally, increased *L. monocytogenes* contamination is detected in fields with more recently cultivated soil and more recent worker activity than the fields with less recent practices [6].

The U.S. Food and Drug Administration (FDA) states that *L. monocytogenes* has been found in raw, unpasteurized milk and cheeses, ice cream, raw or processed vegetables, raw or processed fruits, raw or undercooked poultry, hotdogs, sausages, deli meats, and raw or smoked fish, and even in raw pet food [3]. Meats and dairy products can also contain *L. monocytogenes* because animals may carry the organism without appearing ill or environmental contamination can occur during meat processing [7]. This pathogen has been found on the surface of some raw fruits or vegetables and can also become internalized. Bardsley et al. (2019) reported that, on frozen whole and sliced cucumbers, *L. monocytogenes* can survive for more than 120 d [8]. Thus, it is necessary to evaluate cucumber conditions at each step of the supply chain to reduce potential *Listeria* contamination. Zhu et al. (2017) summarized several studies that reported on the prevalence rates of *L. monocytogenes* in fresh produce, including 21.9% for cucumber [9]. If *L. monocytogenes* contaminates the surface of fresh avocados, then they may enter the avocado pulp from the stem or stem scar. When exposed to the hydrocooled water with *L. monocytogenes*, the pathogen inside the edible parts of avocados can range from 5.90 to 7.19 log CFU/g due to the bacteria’s transmission from the bottom end to the inside of fresh whole avocados [10].

To reduce potential *L. monocytogenes* contamination, the FDA advises that all fruits and vegetables should be washed under running water just before eating, cutting, cooking and peeling. For firm produce such as melons and cucumbers, consumers may need to scrub the surfaces using a clean brush. In addition to using water to remove *L. monocytogenes*, the FDA recommends keeping refrigerated foods under 40°F to reduce risk of *Listeria* infection by slowing down or halting the growth of *L. monocytogenes*. Other prevention measures include cleaning refrigerators frequently to remove food spills, which may harbor *L. monocytogenes,* and washing hands and kitchen surfaces regularly to avoid the cross-contamination of *L. monocytogenes* among different food impact surfaces, such as stainless-steel surfaces used by food-processing industries. In food-processing factories, cleaners can be effectively applied to remove this pathogen on etched stainless-steel surfaces [11].

During the last three decades, cavitation has become a more common treatment methodology to remove bacteria from object surfaces. The definition of cavitation is the formation of vapor cavities, such as bubbles in a liquid, caused by forces acting on the liquid. Cavitation will occur when a rapid change in pressure acts on a liquid to form cavities [12]. Many researchers have studied the cavitation phenomenon and examined the effects of cavitation on industrial systems. Franc and Michel (2010) discuss the history of the study of bubble dynamics, including cavitation [13].

Cavitation and bubble dynamics have a wide range of practical applications in many fields, including hydraulic, mechanical and naval engineering, oil exploration, clinical medicine, sonochemistry, and ultrasonic cleaning for electrical and medical microdevices [14]. In wastewater, hydrodynamic cavitation can successfully remove pharmaceuticals, cyanobacteria, algae, *Legionella* and Rotavirus in an energy-efficient way [15]. The application of physical cavitation bubbles was found to remove and inactivate *Listeria* on the surface of fresh Roma tomatoes and cantaloupes. Lee et al., (2018) reported that *L. monocytogenes* on the surface of fresh Roma tomatoes and cantaloupe was detached and inactivated using a physical continuous bubble stream. The efficacy of the cavitation bubble stream (14 L air/min for 1 min) was 2.89 and 2.63 log pathogen reductions for Roma tomatoes and cantaloupe, respectively [12].

The goal of this research was to evaluate the removal of a biological contaminant from a food or food-contact surface by the shear forces generated from the impact of air bubbles. Therefore, a low-intensity bubble process, with air being injected into water, was developed to determine the efficacy of microbubbles for detaching loosely or firmly attached cells of *L. monocytogenes* from stainless steel (a food-contact surface) and the surface of fresh cucumber and avocado. Moreover, cross-contamination from inoculated to uninoculated cucumbers and avocados was evaluated to determine the level of cross-contamination that may occur. Finally, a quality evaluation was performed on cucumber and avocado after bubble treatment to compare changes in naturally occurring bacterial populations that may lead to microbial spoilage during storage.

## 2. Materials and Methods

### 2.1. Inoculation of Test Surfaces with L. monocytogenes

*L. monocytogenes* (strain LCDC, serotype 4b) was transferred from frozen (−80 °C) storage to Tryptic Soy Broth (TSB) and incubated at 35 ± 2 °C for 24 h. Then, the culture was transferred to fresh TSB and incubated at 35 ± 2 °C for 24 h. This culture was subsequently used to inoculate stainless steel, cucumbers and avocados. Culture identity and purity was confirmed by colony appearance on Rapid L’mono agar (Bio-Rad Laboratories, Richmond, CA, USA) and with the Microgen *Listeria*-ID biochemical identification kit (Microbiology International, Frederick, MD, USA). Additionally, this culture was confirmed to be able to adhere to the test materials and could be recovered 24 and 48 h after inoculation at higher concentrations than three other test strains of *L. monocytogenes*.

Stainless-steel sheet (type 304, #2 Finish; Speedy Metals, Milwaukee, WI, USA) was cut into 1 × 2-inch coupons. Slicing cucumbers (*Cucumis sativas*) and avocados (*Persea americana* cultivar Hass) were purchased from local supermarkets. The degree of maturity for all cucumbers and all avocados was likely similar, since growers and packers had determined they were suitable for distribution. These markets generally sell these produce items within three days after they are made available to the consumer. The Hass avocado is the most common cultivar of commercial avocados. Before the bacterial inoculation onto cucumbers, wax was removed from the cucumber surfaces by washing them with peptone water and drying them gently with paper wipes. Additionally, cucumbers and avocados were sprayed with 70% ethanol and then wiped and air-dried to reduce background microflora.

Stainless-steel coupons were spot inoculated with 0.1 mL of a *L. monocytogenes* culture (~2 × 10^8^ CFU/mL). The spots of inoculation were marked before inoculation. Inoculums were allowed to dry for 1, 24, or 48 h at room temperature (22 °C) in a sealed box with two small beakers of a sodium chloride solution to maintain the humidity from 50% to 60%. Cucumbers and avocados were also spot-inoculated with 0.1 mL of a *L. monocytogenes* culture (~2 × 10^8^ CFU/mL) and stored at 10 °C. Inoculums were allowed to dry for 1, 24, or 48 h.

### 2.2. Bubble Treatments for Stainless Steel

An air compressor (Campbell Hausfeld, Cincinnati, OH, USA, 120 V, 60 Hz, 2 A, 1 gallon tank, maximum rated 110 psi) was used to deliver air through a bubble diffuser (Model MBD75 (Diffuser Area 6-1/8″ × 1-1/8″, Range up to 0.75 LPM, Flow Rate @ 50 PSI 2.2 LPM, Gas Inlet Connection 1/4″ Hose Barb); Pentair, Golden Hills, MN, USA) submerged in the bottom of a water tank. The manufacturer states that this diffuser produces bubbles with an approximately 100–500 micron diameter in water, hereafter referred to as microbubbles. A Masterflex flow meter (Cole-Parmer, Vernon Hills, IL, USA) was used to regulate air flow to 1.0 L/min.

After stainless-steel coupons were dried (1, 24, or 48 h), but before the application of bubble treatments, the inoculated steel coupons were rinsed with 10 mL peptone water to remove loosely attached cells. These rinsates were collected and further analyzed as described in Section 2.4. Stainless steel coupons were individually placed into a 13 L plastic tank containing 4.5 L distilled water. Plastic forceps were used to hold the rectangle stainless-steel coupon when applying bubbles to the inoculated surface.

Stainless-steel coupons were treated for 0, 1, 2, 5 or 10 min using the bubble diffuser with a 1.0 L/min air flow rate. Three steel samples were tested for each combination of inoculum drying time and bubble treatment time. After treatment with a stream of heavy bubbles, stainless-steel coupons were collected in a sterile bag with peptone water and sonicated (Aquasonic Ultrasonic Cleaner, Volts: 117/120, 50/60 Hz) for 2 min at room temperature. Solutions were then diluted with peptone water and plated onto Oxford agar (Neogen, Lansing, MI, USA) plates. Plates were incubated at 35 ± 2 °C for 48 h. Three replications of the above process were conducted for a total of 108 samples.

### 2.3. Bubble Treatment of Cucumber and Avocado Surfaces

After inoculated cucumbers and avocados were dried (1, 24, or 48 h), but before the application of bubble treatments, they were rinsed with 10 mL peptone water to remove loosely attached cells. These rinses were collected and further analyzed as described in Section 2.4. Rinsed cucumbers and avocados were individually placed into a 13 L plastic tank containing 4.5 L distilled water. Two elastic rubber wires on the top the opposite edges of the tank were used to hold the cucumbers in place over the bubble stream. A compressed air supply was used to deliver 1.0 L/min of air through a bubble diffuser as described in Section 2.2. A stream of bubbles (~0.1–0.5 mm diameter) was applied for either 0, 1, 2, 5 or 10 min. Three cucumbers and three avocados were tested for each combination of inoculum drying time (3) and bubble treatment time (5) for each of the three replications.

### 2.4. Recovery of L. monocytogenes from Inoculated Surfaces

Before the application of bubble treatments, inoculated steel coupons, cucumbers and avocados were rinsed with 10 mL distilled water to remove loosely attached cells. The rinses were collected, diluted with peptone water and plated onto Oxford agar plates. Plates were incubated at 35 ± 2 °C for 48 h and enumerated. Then, the rinsed test materials were treated with a bubble application for 0, 1, 2, 5, or 10 min. Firmly attached cells were removed from the steel coupons, cucumbers and avocados by placing the sample in a sterile bag or cup with added peptone water. Containers were then sonicated for 2 min at room temperature (22 °C). Solutions were then diluted and plated onto Oxford agar plates and incubated at 35 ± 2 °C for 48 h. Finally, the cell counts of the solutions after sonication of bubble-treated samples were compared to the cell counts of the solutions after sonication where samples were not treated with bubbles (0 min treatment).

### 2.5. Cross-Contamination from Inoculated to Un-Inoculated Produce

To simulate the cross-contamination of produce with *L. monocytogenes*, one inoculated cucumber and one un-inoculated cucumber were added to a water tank. All cucumbers were wiped to remove wax and reduce background microflora, as described previously. Inoculated cucumbers received 0.1 mL of a *L. monocytogenes* (LCDC) culture and allowed to dry for 1, 24 or 48 h before they were placed into a water tank with one un-inoculated cucumber. A heavy bubble stream from the bubble diffuser was applied for 2 or 10 min with an airflow rate of 1.0 L/min. As a control, pairs of inoculated and uninoculated cucumbers were placed in the water tank for 1 min, but without a bubble application. Then, each sample cucumber was placed into separate sterile bags, and *L. monocytogenes* were recovered from the sample surface, as described previously. Oxford agar plates were incubated at 35 ± 2 °C for 48 h. A similar inoculation, treatment and recovery protocol was carried out for avocados. Three sample pairs of cucumbers and avocados were tested for each combination of inoculum drying time and bubble treatment time. These experiments were replicated three times for both cucumbers and avocados. The proportion of cells that were transferred to the uninoculated samples was determined.

### 2.6. Microbial Quality Evaluation of Treated Cucumbers and Avocados after Refrigerated Storage (10 °C)

Fresh cucumbers and avocados were stored in a refrigerator at 10 °C for up to 7 d. After storage for 0, 1, 4, or 7 d, three cucumbers and three avocados were individually placed in a sterile bag, diluted with peptone water, and solutions were plated onto Tryptic Soy Agar plates. Additional cucumbers and avocados were treated to a heavy bubble stream, as described previously, for 2 or 10 min. These samples were stored for either 0, 1, 4 or 7 d at 10 °C. After storage, three cucumbers and three avocados (from each storage time) were individually placed in a sterile bag, diluted with peptone water, and solutions were plated onto Tryptic Soy Agar plates. All plates were incubated at 35 ± 2 °C for 24 h, then enumerated. The above process was repeated for 3 replications.

### 2.7. Data Analysis

*L. monocytogenes* cell counts recovered from fresh produce and stainless-steel coupons were compared to the cell counts of original inoculation on produce and stainless steel to determine the pathogen detachment from the surface of fresh produce and stainless steel after microbubble treatment. A one-way analysis of variance (ANOVA) was used to determine significant differences between means for each variable tested at a statistical significance of α = 0.05. All calculations were performed with JMP 16.1 Statistical Software (SAS Institute, Inc., Cary, NC, USA).

## 3. Results

### 3.1. Bubble Treatments of Stainless-Steel Coupons

**Figure 1:**
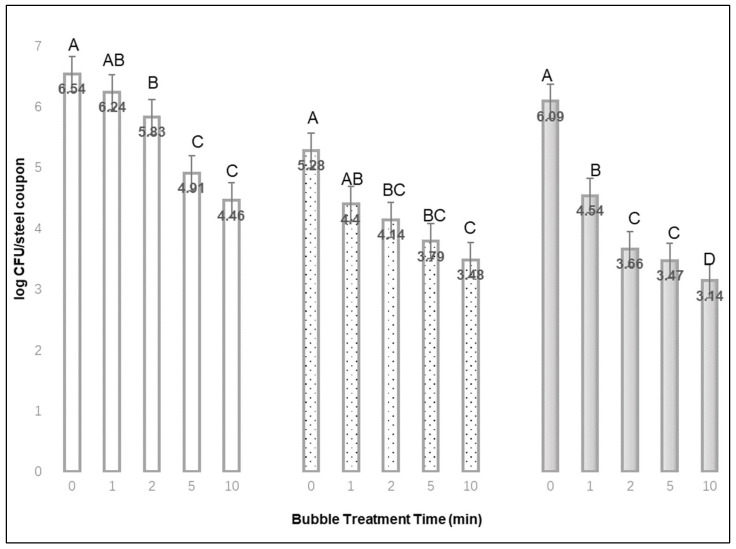
The mean log CFU *Listeria monocytogenes* recovered per stainless steel coupon after inoculum drying times of 1 h 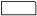, 24 h 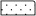, or 48 h 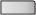, and different bubble treatment times from 0 to 10 min (Mean of: 3 replications × 3 samples each). ^A,B,C,D^ Denote significant differences in recovery (*p* < 0.01) between treatment times for each of the inoculum drying times.

After rinsing off loosely attached cells, approximately 6.65, 6.30, and 6.08 log mean CFU of *L. monocytogenes* per stainless steel coupon could be recovered after 1-, 24-, or 48-h drying time, respectively. As the bubble treatment time increased from 0 to 1, 2, 5, or 10 min, the total mean CFU of *L. monocytogenes* recovered from stainless steel decreased for each set of coupons with different drying times (1, 24, or 48 h). Thus, an extended bubble treatment time appears to remove more bacteria, since less could be recovered from the steel coupons. Figure 1 shows the mean log CFU *L. monocytogenes* recovered per steel coupon after different bubble treatment times, ranging from 0 to 10 min. After inoculated steel was dried for 48 h, the log-CFU-recovered difference (from the no-bubble treatment) was highest after 10 min treatments (2.95 log CFU), and 1.55 log CFU was highest after a 1 min bubble treatment. Additionally, the mean log CFU difference recovered from stainless steel with 1 h drying time was highest after 10 min treatments (2.08 log CFU) and lowest after 1 min treatments (0.31 log CFU). The effect of bubble treatment time was significant for each of the three steel inoculum drying times (*p* < 0.01).

### 3.2. Bubble Treatment of Cucumber Surfaces

**Figure 2:**
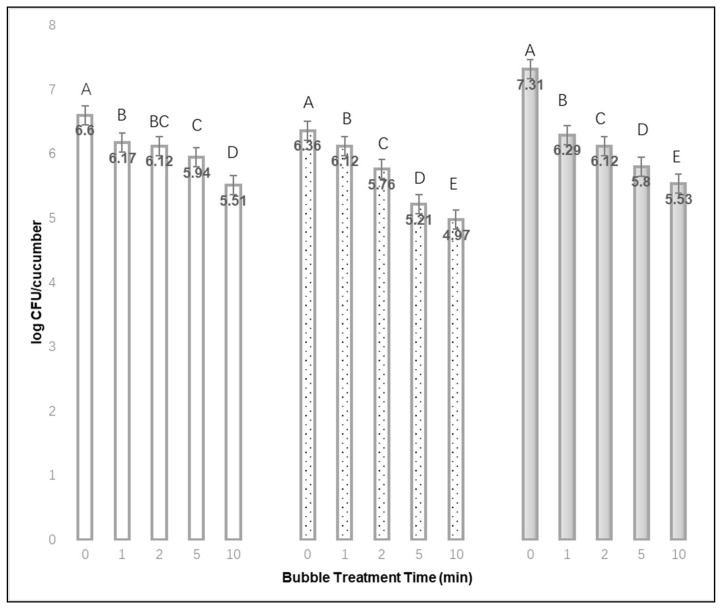
The mean log CFU *Listeria monocytogenes* recovered per cucumber after inoculum drying times of 1 h 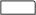, 24 h 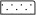, or 48 h 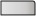, and different bubble treatment times from 0 to 10 min (Mean of 3 replications × 3 samples each). ^A,B,C,D,E^ Denote significant differences in recovery (*p* < 0.01) between treatment times for each of the inoculum drying times.

After rinsing off loosely attached cells, approximately 7.15, 6.48, and 6.79 log mean CFU of *L. monocytogenes* per cucumber could be recovered after a 1, 24, or 48 h drying time, respectively. Figure 2 shows the mean log CFU *L. monocytogenes* recovered per cucumber after rinsing off loosely attached cells, followed by a bubble treatment from 0 to 10 min. The difference in *Listeria* recovery between no-bubble treatments (0 min) and 10 min treatments was 1.09, 1.39 or 1.78 mean log CFU per cucumber for cucumbers dried for 1, 24 or 48 h, respectively. For each of the drying times, extending the bubble treatment times by up to 10 min appears to remove more bacteria, since bacterial recovery from treated cucumbers was reduced. The mean difference in recovery with a 1 min bubble treatment was 1.02 log CFU for cucumbers, where inocula was dried for 48 h, but only 0.24 log CFU when inoculated cucumbers were dried for 24 h. The effect of bubble treatment time was significant for each of the cucumber drying times (*p* < 0.01). When cucumbers were dried for 24 or 48 h, the bacterial recovery of firmly attached cells was significantly different (lowered) between each of the bubble treatment times (*p* < 0.01).

### 3.3. Bubble Treatment of Avocado Surfaces

After rinsing off loosely attached cells, approximately 8.13, 7.52, and 7.79 log mean CFU of *L. monocytogenes* per avocado could be recovered after 1, 24, or 48 h drying time, respectively. As the bubble treatment time increased from 0 to 1, 2, 5, or 10 min, the mean CFU of *L. monocytogenes* recovered from avocados decreased for each set of fruit with different drying times (1, 24, or 48 h). Therefore, extended bubble treatment times may lead to greater bacterial removal or detachment.

**Figure 3:**
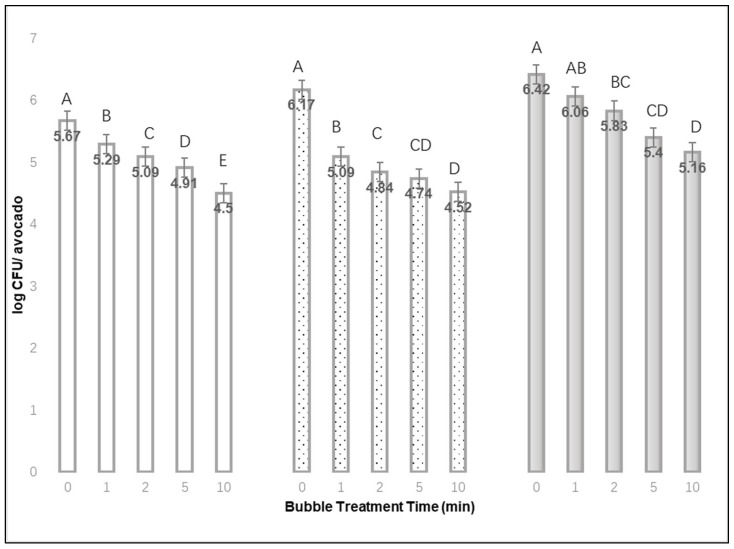
The mean log CFU *L. monocytogenes* recovered per avocado after inoculum drying times of 1 h 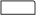, 24 h 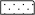, or 48 h 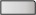, and different bubble treatment times from 0 to 10 min (mean of 3 replications × 3 samples each). ^A,B,C,D,E^ Denote significant differences in recovery (*p* < 0.01) between treatment times for each of the inoculum drying times.

Figure 3 shows the mean log CFU *L. monocytogenes* recovered per avocado after different bubble treatment times, ranging from 0 to 10 min. After a 1 min treatment, the mean difference (from 0 min treatment) log CFU recovered from avocado with a 24 h drying time (1.08 log CFU) was greater than that for the avocados that were dried for 1 or 48 h. The maximum difference from a no-bubble treatment occurred with a 10 min treatment for avocadoes where the inoculum was dried for 24 h (mean 1.65 log CFU per avocado). Similar to cucumbers, the effect of bubble treatment time was significant for each of the cucumber drying times (*p* < 0.01). When avocados were dried for 1, 24 or 48 h, the bacterial recovery of firmly attached cells was significantly different between most of the bubble treatment times (*p* < 0.01).

### 3.4. Inoculated to Un-Inoculated Produce Cross-Contamination Simulations

#### 3.4.1. Cucumber

Table 1 shows the percent transfer of viable *L. monocytogenes* cells from cross-contaminated cucumbers with different bubble treatment times and the three different inocula drying times. The percent transfer from contaminated cucumbers to uninoculated cucumbers increased when bubble treatment time increased. For 2 and 10 min bubble treatments, the percent transfer of *L. monocytogenes* (0.203% and 0.548%) between cucumbers was the highest from inoculated cucumbers dried for 24 h compared to those dried for 1 or 48 h. When pairs of cucumbers were placed in water for 1 min and without a bubble treatment, no more than 0.002% of the recovered *L. monocytogenes* was transferred to uninoculated cucumbers.

#### 3.4.2. Avocado

Table 1 also shows that the percent transfer from cross-contaminated avocados increases when bubble treatment time increases, but at a lower level than seen with cucumbers. For 2 and 10 min bubble treatments, the percent transfer of *L. monocytogenes* between avocados (0.017% and 0.115%, respectively) was highest from inoculated avocados dried for 24 h than for 1 or 48 h. When pairs of avocados were placed in water for 1 min without a bubble stream, no more than 0.001% of the recovered *L. monocytogenes* was transferred to uninoculated avocados.

### 3.5. Quality Evaluation of Produce during Refrigerated Storage

#### 3.5.1. Cucumber

**Figure 4:**
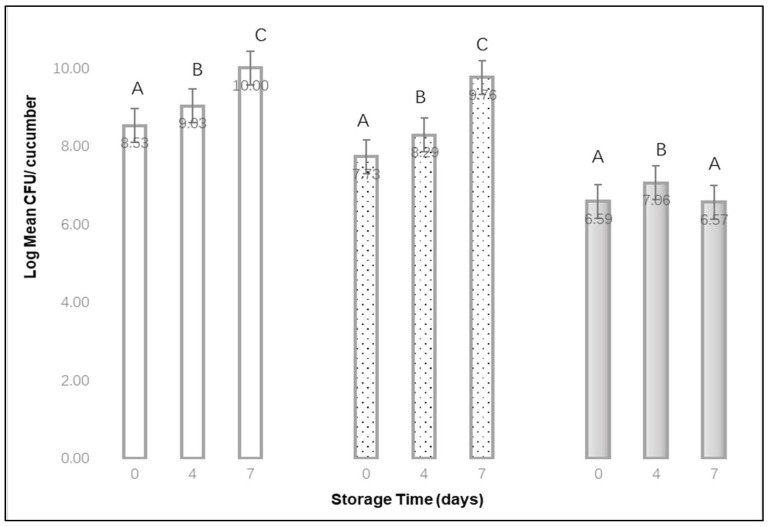
Log Mean CFU per cucumber recovered after different storage times, at 10 °C, when treated with a 0 min 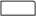, 2 min 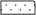, or 10 min 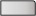 bubble stream. (mean of 3 replications × 3 samples each). ^A,B,C^ Denote significant differences in recovery (*p* < 0.01) between storage times for each of the bubble treatment times.

Figure 4 displays the log mean CFU per cucumber recovered after different storage times after different bubble treatment times. When no bubbles were applied (0 min) and for a 2 min bubble treatment time, the log mean CFU per cucumber increased from day 0 to day 7. For 10 min bubble treatment, log mean CFU per cucumber increased from day 0 to storage day 4, then decreased slightly by storage day 7. Overall, the log mean CFU per cucumber after 0, 4 and 7 d storage was the lowest when bubbles were applied for 10 min.

#### 3.5.2. Avocado

**Figure 5:**
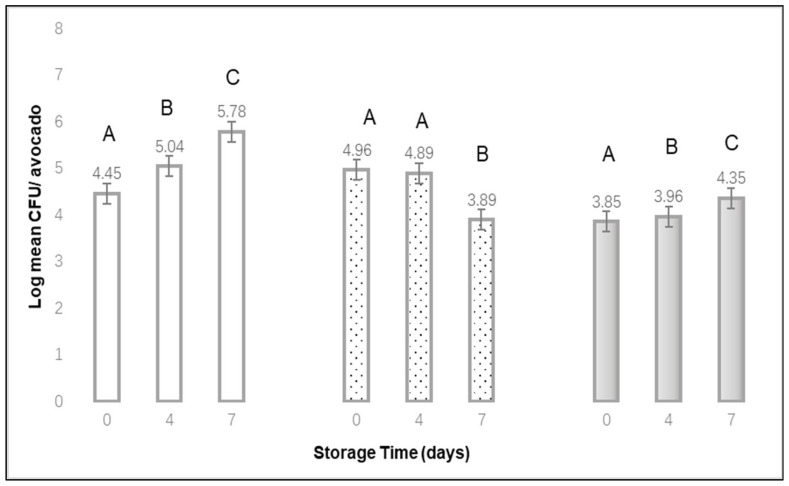
Log Mean CFU per avocado recovered after different storage times, at 10 °C, when treated with a 0 min 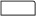, 2 min 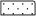, or 10 min 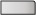 bubble stream (mean of 3 replications × 3 samples). ^A,B,C^ Denote significant differences in recovery (*p* < 0.01) between storage times for each of the bubble treatment times.

As seen in Figure 5, the log means CFU per avocado increased during storage from day 0 to day 7 when avocados received no bubble treatment or when they received a 10-min bubble treatment. For the 2 min’ bubble treatment, the log mean CFU per avocado decreased by approximately 1 log after 7 d storage, yet almost no decrease was observed after 4 d storage. The mean log CFU recovered immediately after a 2 min bubble treatment (log 4.96 for day 0), which was higher than the initial count recovered for the no-bubble treatment or 10 min treatment (4.45 or 3.85 log CFU per avocado, respectively).

## 4. Discussion

Based on this study, a stream of targeted by microbubbles (<0.5 mm dia.) from a bubble diffuser (1.0 L air/min) can detach cells of *L. monocytogenes* from steel and produce surfaces. Extended bubble treatment times (up to 10 min) promoted greater removal of *L. monocytogenes* from stainless steel coupons, since significantly fewer *L. monocytogenes* were recovered from stainless steel after 10 min bubble treatments compared with no bubble treatment. The bubble application used in this study may reduce *L. monocytogenes* on stainless by nearly three logs (2.95 log CFU per sample) after an inoculum was dried for 48 h and loosely attached cells were removed. For cucumber and avocado, a 5 min application of the same heavy bubble stream removed at least 1.0 log more of the firmly attached bacteria than submerging the produce in water without bubbles.

Lee et al. (2018) applied cavitating bubbles to inoculated fresh Roma tomatoes and cantaloupes using larger bubble sizes (~1–3 mm diameter) and higher air flows (3.5–14 L/min) than were used in this study [12]. Those authors reported that *L. monocytogenes* significantly decreased on Roma tomatoes (~1.0 log CFU after 30 s and ~1.2 log CFU after 60 s treatment) with a 14 L/min airflow rate compared to no cavitating bubbles. *L. monocytogenes* significantly decreased on cantaloupes (0.65 log CFU for 30 s and ~0.74 log CFU for 60 s treatment) when the maximum air-flow rate was used. In the study reported here, the log reduction in *L. monocytogenes* on cucumber and avocado surfaces also increased with increasing bubble treatment time, even though the airflow rate was lower, the treatment times longer, and the bubble diameters smaller. An important difference in these studies is that Lee et al. (2018) did not remove loosely attached cells prior to any bubble treatments. Here, in our study with cucumbers, avocados and stainless-steel coupons, the number of loosely attached cells recovered and enumerated after 1, 24 or 48 h drying was always higher than the quantity that could be recovered from the samples that were not treated with bubbles (treatment time = 0 min).

Chlorine is commonly applied to wash and clean fresh produce due to its bactericidal capacity and economical cost. When chlorine makes contact with wash water during washing, it yields an oxidizer, HOCI, which can inactivate pathogens effectively [16]. The common dosage of commercial chlorine in industry ranges from around 50 to 200 mg/L, but 100 mg/L is the most common dosage used in industry [17]. The application of this dosage of chlorine requires a short impact time, of about 1 to 2 min, and a preferred pH of from 6.0 to 7.5 is used to maintain the stabilization of HOCI in order to prevent chemicals from corroding the processing equipment. Banach et al. (2015) showed that a five-log CFU of *E. coli* on fresh spinach can be completely removed with wash water containing a level of 7 mg/L free chlorine [16]. Although some chlorine solutions have been shown to inactivate pathogens at a higher level than reported in the present study using streams of microbubbles, some regulators, consumers, and food industries believe that an over-reliance on chlorine rinses by food industries can lead to the excessive formation of chlorate residues that can harm human health.

Other researchers have compared the addition of several antimicrobial chemicals to the treatment water of fresh produce regarding their ability to inactivate or reduce pathogens, including *Listeria monocytogenes*. Chlorine, peracetic acid (PAA), chlorine dioxide (CIO_2_), ozone, and electrolyzed oxidizing water (EOW) are some of the most common antimicrobial agents [18]. Rodgers et al. (2004) showed that both *E. coli* O157:H7 and *L. monocytogenes* can be inactivated over five logs when exposed to aqueous chemical sanitizers, including ozone, ClO_2_, chlorine, and PAA, for a 2–5 min exposure time [19]. Additionally, ozonation can be an excellent alternative for chlorine, as an economical and environmentally friendly disinfectant for this industry [20]. Additionally, ultraviolet light energy could be an effective measure by which industry could clean fresh produce. Yaun et al. (2004) reported using ultraviolet energy (24 mW/cm^2^) to remove *Salmonella* spp. or *Escherichia coli* on the surface of fresh produce, including red apples, leaf lettuce, and tomatoes. These researchers reported that both *Salmonella* (2.65 log reduction) and *E. coli* (2.79 log reduction) can be inactivated to a similar level [21].

The present study was designed to evaluate microbubbles for removal or detachment of *L. monocytogenes* on stainless steel, avocado and cucumber surfaces. Even though microbubbles may reduce the concentration of *L. monocytogenes* on steel and some produce surfaces, they may or may not enhance microbial inactivation. Nevertheless, microbubbles can be an environmentally friendly alternative to chlorine for reducing the microbial surface contamination of fresh produce, which will be odorless and possibly less corrosive to equipment.

Based on the results, the cross-contamination of *L. monocytogenes* from inoculated to clean cucumbers and avocados increased with extended bubble treatment time (2 vs. 10 min). The transfer rate to cucumbers and avocados was also higher when inoculum was dried for 24 h versus 1 or 48 h. This result could be explained by differences in the total number of firmly or loosely attached cells that occurred with each drying time. The variable numbers of attached cells on inoculated cucumbers and avocados influence the number of cells that detach and adhere to uninoculated produce. The percentage of cells that were transferred between cucumbers was always higher than the percentage transfer between avocados, even though the initial inoculums were similar. Compared with cucumbers, the cross-contamination of avocados by *L. monocytogenes* was less affected by bubble treatments. In Lee et al. (2018), the cross-contamination of *L. monocytogenes* on Roma tomatoes was not significantly different with bubble treatment time (30 or 60 s) in terms of the transfer between inoculated to uninoculated Roma tomatoes, but significantly different between airflow rates (0, 7 or 14 SLPM) [12].

The application of a bubble stream similar to that used in this study could be used in a produce-packing house prior to the packaging and shipment of some produce to retail markets. This technique is not likely to be employed by consumers due to the need to purchase equipment that will be only occasionally used. After the consumer’s retail purchase, the shelf-life of fresh, whole cucumbers is approximately 7 d when refrigerated (1–10 °C), and the shelf-life of fresh avocados is ~3–4 d at room temperature (20–25 °C) and ~7–10 d when refrigerated [22,23]. In this study, extending refrigerated storage from 4 to 7 d resulted in variable increases or decreases in surface bacteria growth and changes in quality for cucumbers and avocados. While an extended bubble treatment time appears to have higher effectiveness for removing bacteria from cucumbers, the surface aerobic plate count concentrations did not decrease over time. Similarly, for avocados, surface aerobic plate counts increased with longer storage times, and surface concentrations could initially be reduced by a bubble treatment, by less than one log CFU per unit. Extended bubble treatment times (>10 min, for example) for raw fruit or vegetables could damage the surface or skin in such a way that bacteria are able to infiltrate. Additionally, submerging some produce in water without bubbles could lead to a disruption in the integrity of the skin or surface. For this project, we did not microscopically examine the physical surface of the test produce.

The efficacy required by a bubble stream to detach bacteria from an irregular surface will vary according to the speed at which a bubble moves through water, its impact angle with a surface and the forces exerted on a surface. Microbubble applications can be more effective for surface cleaning if the angle of contact between the bubbles and the surface and the shear stress exerted on a surface can be measured and controlled [24,25]. Therefore, variations in the distance a bubble travels before surface contact, contact angles, total bubble volume or air-flow rate used, as well as the bubble diameter range, can be adjusted and compared to further characterize a non-thermal aeration process to enhance food safety.

We demonstrated that a stream of injected air bubbles can effectively reduce the population of *L. monocytogenes* on stainless steel, raw cucumbers and raw avocados. As the bubble treatment times increases, more of these bacteria can be removed from individual fruits, but there is a risk of increased bacterial transfer to other nearby fruits. While extended bubble treatment times appear to have higher effectiveness for removing aerobic bacteria populations from cucumbers and avocados, these populations did not further decrease over 7 d of refrigerated storage.

Additional research is needed to understand and optimize an injected air-bubble treatment for raw produce that can consistently, and significantly, reduce surface microbial concentrations. More specific to the study reported here, the detachment of other *Listeria* serotypes or other bacterial pathogens associated with raw fruits or vegetables could be studied. Additionally, the application of a bubble stream may enhance the removal of non-pathogenic or spoilage organisms, which could increase shelf-life under some storage conditions. In addition to cucumbers and avocados, the surface microbial concentrations of melons, tomatoes and papaya could be evaluated, for example, since a consumer could either eat the outer surfaces, or the surface bacteria could be transferred to interior edible portions during slicing.

## Figures and Tables

**Table 1:** *Listeria monocytogenes* transfer from inoculated, dried and rinsed cucumbers or avocados to uninoculated fruit (*n* = 3).

Inocula Drying Time (h)	Air Flow (L/min)	Treatment Time (min)	% Transfer Cucumbers	% Transfer Avocados
1	0	1	0.001%	0.000%
24	0	1	0.002%	0.001%
48	0	1	0.002%	0.001%
1	1	2	0.059%	0.006%
24	1	2	0.203%	0.017%
48	1	2	0.107%	0.012%
1	1	10	0.306%	0.046%
24	1	10	0.548%	0.115%
48	1	10	0.436%	0.092%

## Data Availability

The data that support the findings of this study are available from the corresponding author upon reasonable request.

## References

[1] Interagency Food Safety Analytics Collaboration (2021). Foodborne Illness Source Attribution Estimates for 2019 for *Salmonella*, *Escherichia coli* O157, *Listeria monocytogenes*, and *Campylobacter* Using Multi-Year Outbreak Surveillance Data, United States. U.S. Department of Health and Human Services, CDC, FDA, USDA/FSIS. https://www.cdc.gov/foodsafety/ifsac/annual-reports.html.

[2] CDC Listeria (*Listeriosis*). https://www.cdc.gov/listeria/risk.html.

[3] FDA Listeria (*listeriosis*). U.S. Food and Drug Administration. https://www.fda.gov/food/foodborne-pathogens/Listeria-listeriosis.

[4] Ivanek R., Gröhn Y.T., Tauer L.W., Wiedmann M. (2005). The cost and benefit of *Listeria monocytogenes* food safety measures. Crit. Rev. Food Sci. Nutr..

[5] Hamon M., Bierne H., Cossart P. (2006). *Listeria monocytogenes*: A multifaceted model. Nat. Rev. Microbiol..

[6] Strawn L.K., Gröhn Y.T., Warchocki S., Worobo R.W., Bihn E.A., Wiedmann M. (2013). Risk factors associated with *Salmonella* and *Listeria monocytogenes* contamination of produce fields. Appl. Environ. Microbiol..

[7] FSIS *Listeria* *monocytogenes*. https://www.fsis.usda.gov/inspection/compliance-guidance/microbial-risk/listeria-monocytogenes.

[8] Bardsley C.A., Truitt L.N., Pfuntner R.C., Danyluk M.D., Rideout S.L., Strawn L.K. (2019). Growth and survival of *Listeria monocytogenes* and *Salmonella* on whole and sliced cucumbers. J. Food Prot..

[9] Zhu Q., Gooneratne R., Hussain M. (2017). *Listeria monocytogenes* in fresh produce: Outbreaks, prevalence and contamination levels. Foods.

[10] Chen Y., Evans P., Hammack T.S., Brown E.W., Macarisin D. (2016). Internalization of *Listeria monocytogenes* in whole avocado. J. Food Prot..

[11] Krysinski E.P., Brown L.J., Marchisello T.J. (1992). Effect of cleaners and sanitizers on *Listeria monocytogenes* attached to product contact surfaces. J. Food Prot..

[12] Lee J., Eifert J., Jung S., Strawn L. (2018). Cavitation bubbles remove and inactivate *Listeria* and *Salmonella* on the surface of fresh Roma tomatoes and cantaloupes. Front. Sustain. Food Syst..

[13] Franc J.-P., Michel J.-M. (2005). Fundamentals of Cavitation.

[14] Blake J.R., Leppinen D.M., Wang Q. (2015). Cavitation and bubble dynamics: The kelvin impulse and its applications. Interface Focus.

[15] Dular M., Griessler-Bulc T., Gutierrez-Aguirre I., Heath E., Kosjek T., Krivograd Klemenčič A., Kompare B. (2016). Use of hydrodynamic cavitation in (waste) water treatment. Ultrason. Sonochem..

[16] Banach J.L., Sampers I., Van Haute S., Van der Fels-Klerx H.J. (2015). Effect of disinfectants on preventing the cross-contamination of pathogens in fresh produce washing water. Int. J. Environ. Res. Public Health.

[17] Baur S., Klaiber R.G., Kobli B., Carle R. (2004). Effect of different washing procedures on phenolic metabolism of shredded, packaged iceberg lettuce during storage. J. Agric. Food Chem..

[18] Pietrysiak E., Smith S., Ganjyal G.M. (2019). Food safety interventions to control *Listeria monocytogenes* in the fresh apple packing industry: A review. Comp. Rev. Food Sci. Food Saf..

[19] Rodgers S.L., Cash J.N., Siddiq M., Ryser E.T. (2004). A comparison of different chemical sanitizers for inactivating *Escherichia coli* O157:H7 and *Listeria monocytogenes* in solution and on apples, lettuce, strawberries, and cantaloupe. J. Food Prot..

[20] Rosenblum J., Ge C., Bohrerova Z., Yousef A., Lee J. (2012). Ozonation as a clean technology for fresh produce industry and environment: Sanitizer efficiency and wastewater quality. J. Appl. Microbiol..

[21] Yaun B.R., Sumner S.S., Eifert J.D., Marcy J.E. (2004). Inhibition of pathogens on fresh produce by ultraviolet energy. Int. J. Food Microbiol..

[22] Cucumbers—How Long Do Cucumbers Last?. https://www.eatbydate.com/vegetables/fresh-vegetables/how-long-do-cucumbers-last-shelf-life-expiration-date.

[23] Boyer R.R., McKinney J.M. (2018). Food Storage Guidelines for Consumers.

[24] Menesses M., Belden J., Dickenson N., Bird J. (2017). Measuring a critical stress for continuous prevention of marine biofouling accumulation with aeration. Biofouling.

[25] Hooshanginejad A., Sheppard T., Xu P., Manyalla J., Jaicks J., Esmaili E., Jung S. (2022). Removing proteins or bacteria on a tilted surface using air. arXiv.

